# Reversibility of clinical and computed tomographic lesions mimicking pulmonary fibrosis in a young cat

**DOI:** 10.1186/s12917-021-03081-8

**Published:** 2021-12-09

**Authors:** Alba Stavri, Isabelle Masseau, Carol R. Reinero

**Affiliations:** 1 Department of Veterinary Medicine and Surgery, University of Missouri, 900 E Campus Dr, Columbia, MO USA; 2 Department of Sciences Cliniques, Faculté de Médecine Vétérinaire, Université de Montréal, St-Hyacinthe, Canada

**Keywords:** Computed tomography, Advanced thoracic imaging, Histopathologic correlates, *Toxocara cati*, Idiopathic pulmonary fibrosis, Parasitic lung disease

## Abstract

**Background:**

In humans with idiopathic pulmonary fibrosis (IPF), specific thoracic computed tomographic (CT) features in the correct clinical context may be used in lieu of histologic examination. Cats develop an IPF-like condition with similar features to humans. As few cats have invasive lung biopsies, CT has appeal as a surrogate diagnostic, showing features consistent with architectural remodeling supporting “end-stage lung”.

**Case presentation:**

A 1-year-old female spayed Domestic Shorthair cat presenting with progressive respiratory clinical signs and thoracic CT changes (reticular pattern, parenchymal bands, subpleural interstitial thickening, pleural fissure thickening, subpleural lines and regions of increased attenuation with traction bronchiectasis and architectural distortion) consistent with reports of IPF was given a grave prognosis for long-term survival. The cat was treated with prednisolone, fenbendazole, pradofloxacin and clindamycin. Five months later, while still receiving an anti-inflammatory dose of prednisolone, the cat was re-evaluated with owner-reported absent respiratory clinical signs. Thoracic CT demonstrated resolution of lung patterns consistent with fibrosis.

**Conclusions:**

Fibrotic lung disease is irreversible. Despite this cat having compatible progressive respiratory signs and associated lung patterns on thoracic CT scan, these abnormalities resolved with non-specific therapy and time, negating the possibility of IPF. While the cause of the distinct CT lesions that ultimately resolved was not determined, infection was suspected. Experimental *Toxocara cati* infection shows overlapping CT features as this cat and is considered a treatable disease. Improvement of CT lesions months after experimental heartworm-associated respiratory disease in cats has been documented. Reversibility of lesions suggests inflammation rather than fibrosis was the cause of the thoracic CT lesions. This cat serves as a lesson that although thoracic CT has been advocated as a surrogate for histopathology in people with IPF, additional studies in cats are needed to integrate CT findings with signalment, other clinicopathologic features and therapeutic response before providing a diagnosis or prognosis of fibrotic lung disease.

## Background

Idiopathic pulmonary fibrosis (IPF) is a fatal disease in humans defined as a clinical syndrome of progressive lung scarring with a specific histologic pattern of fibrosis called usual interstitial pneumonia (UIP) [1]. Years of multidisciplinary collaboration between pulmonologists, radiologists and pathologists in the human medical field has led to development of high-resolution CT criteria for a UIP pattern [1]. Collectively, compatible clinical and computed tomographic (CT) features provide a surrogate diagnosis of IPF without need for invasive lung biopsy [1, 2]. Cats develop an IPF-like condition including histologic evidence of UIP with a similarly grave prognosis, reflective of an end-stage fibrotic lung [3]. Lung biopsy, while required for definitive diagnosis of fibrotic lung disease and other disease mimics, is considered an aggressive and invasive diagnostic in cats, and is seldom performed. As in humans, using CT patterns consistent with pulmonary fibrosis as a surrogate for lung biopsy in cats is appealing. While there is a paucity of CT data supported by histologic evaluation for fibrotic lung disease in cats in the literature, advances have been made in dogs and applied to cats. Caution must be applied in this approach as aside from differences between dogs and cats, not all fibrotic lung disease in small animals is IPF (UIP) [4], despite widespread use of this term. Fibrosis represents the end stage pathway for many different insults, and, depending on the underlying trigger and stage, can have varied anatomic appearances on imaging studies and histopathology. Finally, also resulting from lack of CT-histologic correlates in the cat, it is unknown if there are diseases with CT lung patterns considered diagnostic for fibrotic lung disease that are reversible.

This case report describes a young cat with severe respiratory clinical signs and thoracic CT lung patterns consistent with IPF, made by multidisciplinary collaboration between board-certified small animal internal medicine and diagnostic imaging specialists. Clinical signs and CT patterns resolved with empiric therapy and time. Without multidisciplinary collaboration to ensure rigorous correlation of thoracic CT lung patterns with clinical data and histopathology in cats, incorrect assumptions of final diagnosis can be made. While extrapolation of imaging findings from humans or other animal species can serve as a starting point for diagnosis of lung disease in cats, this case suggests caution is warranted in using thoracic CT as a surrogate for histologic confirmation until further studies are performed. As illustrated herein, CT lung patterns previously considered end-stage may be reversible if the underlying disease is treatable.

## Case presentation

**Fig. 1:**
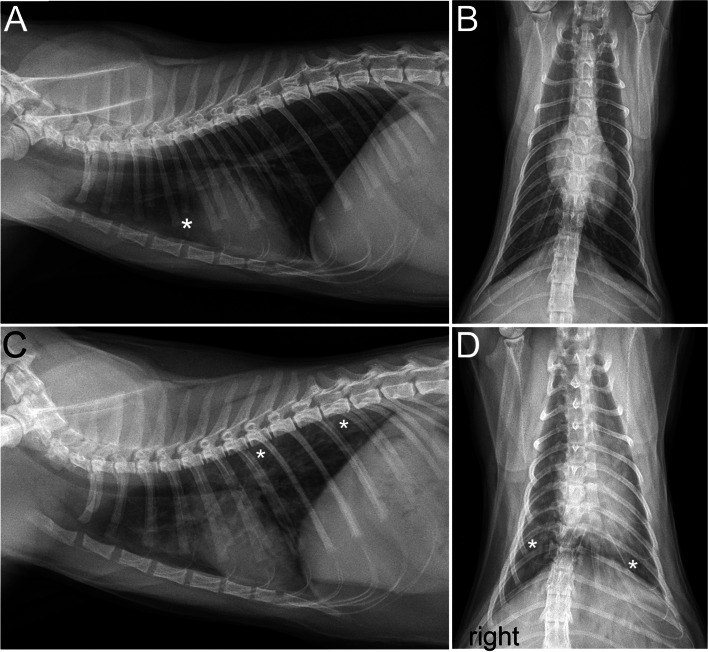
Two sets of thoracic radiographs performed 2 months apart in a 1-year old cat presenting for progressive respiratory disease. Initial lateral (**A**) and ventrodorsal (**B**) projections provided by the primary care veterinarian showing an unremarkable lung field. The opacity (*) cranial to the cardiac silhouette was attributed to thymus and/or fat in the mediastinum. Lateral (**C**) and ventrodorsal (**D**) projections were repeated 2 months later following worsening of respiratory signs. A diffuse bronchointerstitial pattern with multifocal discrete patchy areas of alveolar pattern (*) is now seen

A 1-year-old female spayed Domestic Shorthair (3.55 kg) presented to the University of Missouri Veterinary Health Center (VHC) for severe tachypnea and wheezing. She was acquired from a shelter 2 months earlier with a history of respiratory disease. She had been treated for presumptive pneumonia with unknown antibiotics by the shelter veterinarian. After adoption, the cat was seen by the primary care veterinarian and treated for intermittent hyporexia, increased respiratory effort and rate, wheeze and cough. Over several visits, various medications at dosages not recorded in the medical record were administered including oral antibiotics (amoxicillin, amoxicillin/clavulanate and doxycycline), nebulized gentamycin, injectable methylprednisolone, oral prednisolone and a single dose of praziquantel/pyrantel (Drontal; Bayer DVM). She was vaccinated for feline viral rhinotracheitis, calicivirus and paneleukopenia. Repeated CBC and chemistry panels found no clinically relevant abnormalities such as a peripheral eosinophilia. Serial thoracic radiographs showed progression to a bronchointerstitial and patchy alveolar pattern (Fig. 1). Minimal sustained response to treatments were observed, prompting referral to the VHC.

Tachypnea (140 breaths per minute) and increased bronchovesicular sounds were noted by the VHC emergency veterinarian. Thoracic ultrasonography identified B-lines prompting treatment for presumptive congestive heart failure with furosemide (4 mg/kg IM then 2 mg/kg IV q6h for 2 additional doses), butorphanol (0.3 mg/kg IM once) and supplemental oxygen. An echocardiogram was performed; findings suggested probable pseudohypertrophy of the left ventricle and a volume-underloaded heart. Testing for FeLV, FIV and heartworm antigen were negative. CBC was consistent with hemoconcentration with no other abnormalities. A renal panel identified mild azotemia. Oxygen therapy was continued and furosemide was discontinued; IV fluid therapy with Plasma-Lyte 148 was initiated (47 mL/kg/d).

**Fig. 2:**
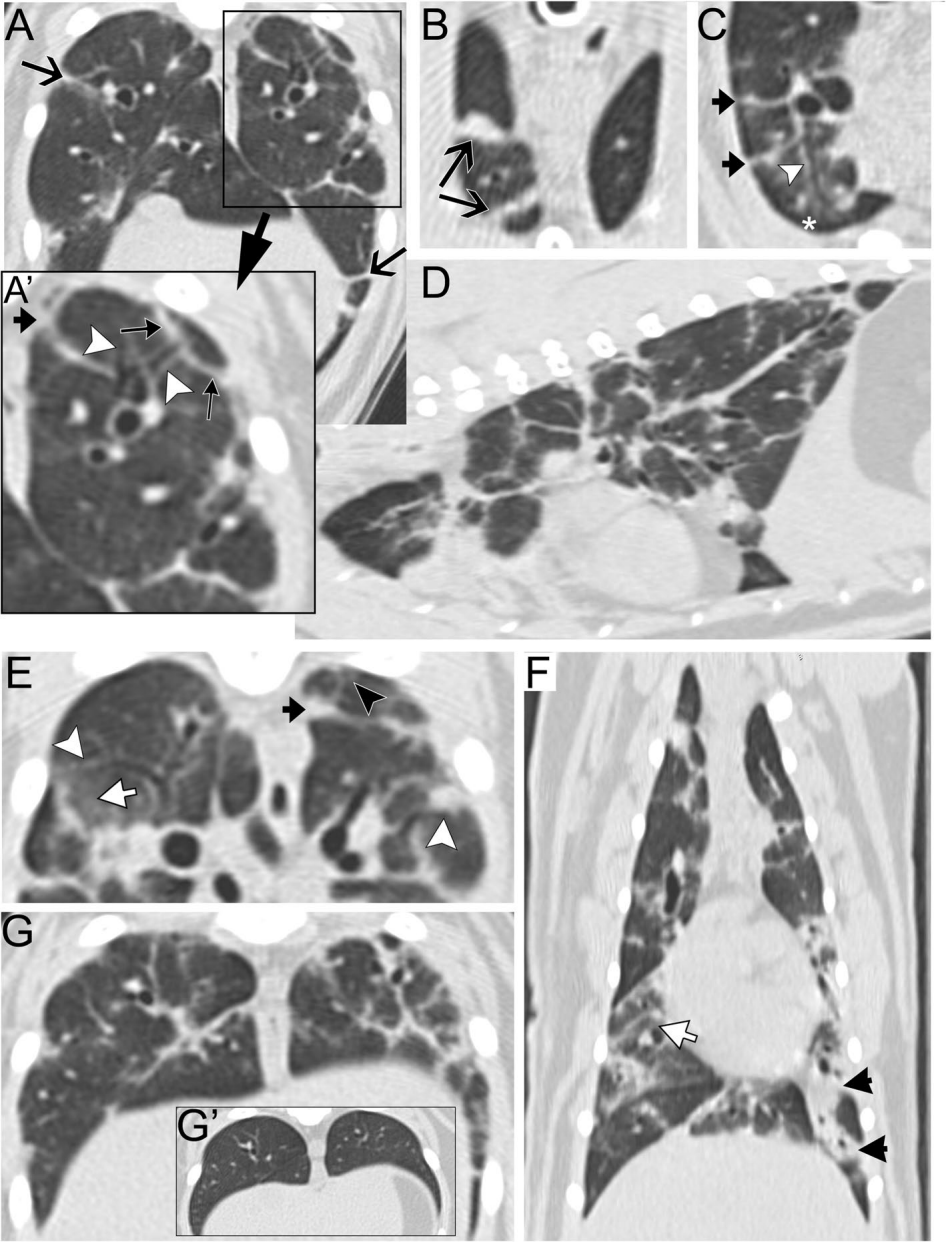
Computed tomographic (CT) inspiratory images in a one-year old cat with progressive respiratory signs. The CT images (**A**-**G**) were acquired 2 days after the second set of radiographic images (shown in Fig. 1C-D) except for G’ (inset) depicting the same region as in G, but 5 months later. Linear opacities were the predominant lesions followed by parenchymal opacification lesions and both categories were observed multifocally throughout the entire length of the thorax, as shown on the multiplane reconstruction sagittal (**D**) and dorsal (**F**) images. (A, inset (A’)) Numerous parenchymal bands (V-shaped arrows), subpleural lines (triangle-shaped arrows) and subpleural interstitial thickening were seen. (**B**) Parenchymal bands varied in thickness and were predominantly located at the periphery of the lungs, some of them contacting both mediastinal and costal surfaces of the pleura (V-shaped arrows). (**C**) Subpleural interstitial thickening (black arrows) occasionally summated with vessels. (A, A’, C, E) Bronchovascular bundle thickening (white arrowheads) and tree-in-bud pattern (black arrowhead) were seen multifocally often with non-tapering of small airways (*). Parenchymal opacification consisting of ground glass opacity (white arrows in **E** and **F**) and a few areas of consolidation (black arrows in **F**) were present multifocally. Some of the linear opacities were difficult to differentiate from thickened bronchovascular bundles as seen in G. Comparison with a CT image of the same level acquired 5 months later allows to appreciate the extent of the linear opacities initially present and their subsequent resolution

The following day after transfer to the internal medicine department, fecal floatation and Baermann failed to identify parasites. Anesthesia was performed with butorphanol (0.5 mg/kg IV) for premedication, followed by propofol (6 mg/kg IV) for induction. Anesthetic maintenance was performed using a propofol constant rate infusion (0.3 mg/kg/min). A thoracic CT study with applied ventilator-assisted inspiratory and expiratory breath-holds and single-phase angiography was performed (Fig. 2). The predominant lesions were linear opacities (reticular abnormalities, parenchymal bands, subpleural lines and subpleural interstitial thickening) followed by increased attenuation (ground glass opacity and a few areas of complete opacification). Both categories were observed multifocally throughout the entire length of the thorax. At the time of interpretation, findings were interpreted as consistent with idiopathic pulmonary fibrosis. While not initially noted, subsequent review found evidence of bronchiolar disease with tree-in-bud pattern and bronchiolectasis (non-tapering of small airways) [5]. Bronchoalveolar lavage (BAL) revealed 39% large mononuclear cells, 37% non-degenerate neutrophils, 14% small mature lymphocytes, 10% eosinophils with occasional globule leukocytes. BAL culture yielded no growth. Oral prednisolone (1.4 mg/kg PO q24h) was prescribed to treat the inflammation identified by BAL, and pradofloxacin (7 mg/kg PO q24h [Veraflox; Bayer DVM]) was prescribed to empirically treat for *Mycoplasma* species due to a lack of prior response to doxycycline. Fenbendazole (45 mg/kg PO q24h PO for 5 days [Panacur; Merck Animal Health]) was prescribed to empirically treat for *Aelurostrongylus abstrusus*, *Toxocara cati* or other helminths. This was prescribed since a negative fecal flotation and Baermann examination do not confidently rule out these diseases. *Toxoplasma* IgG and IgM titers returned 6 days later; the cat was IgG positive (1:2560) and IgM negative (< 1:40). The significance of the single high IgG titer was unclear, but in light of clinical signs and treatability of *T. gondii*, the antimicrobial was switched to clindamycin (9.9 mg/kg PO q12h for 30 days).

Phone conversation with the owner 1-month post-discharge revealed that the patient was doing well with very intermittent coughing. The prednisolone dose was decreased to 1.1 mg/kg PO q24 6 weeks after discharge. Further dose reduction (0.84 mg/kg PO q24h) occurred after 6 additional weeks. Presumptive bacterial cystitis was treated by her primary care veterinarian using cefovecin (8.4 mg/kg SC [Convenia; Zoetis US]), amoxicillin/clavulanate (dose unknown [Clavamox; Zoetis US]) and buprenorphine (dose unknown) at 2 separate visits.

**Fig. 3:**
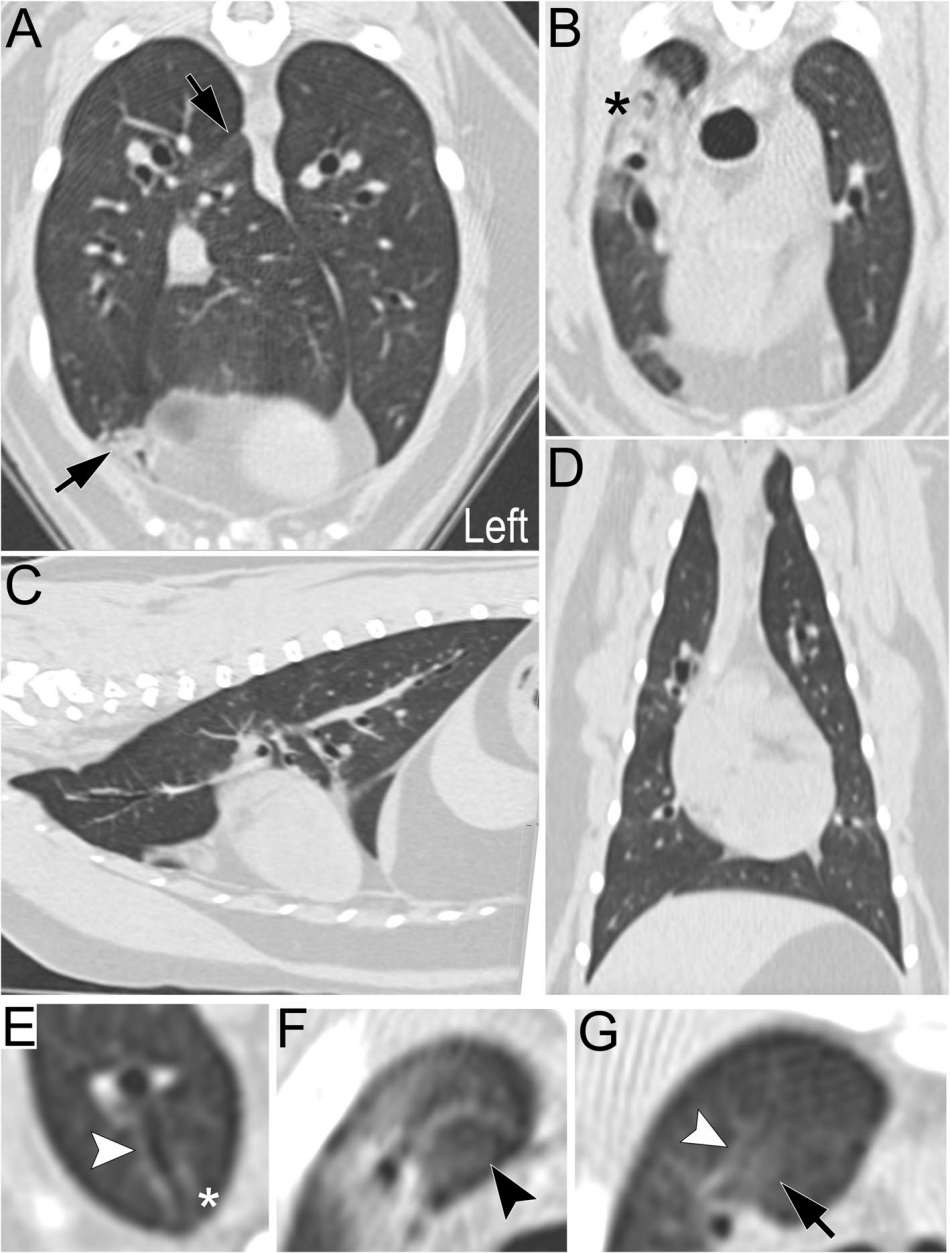
Computed tomographic thoracic images of the same cat as in Fig. 1 acquired 5 months later. Most linear and reticular linear opacities have resolved as shown on the transverse (**A**-**B**), sagittal (**C**) and dorsal (**D**) images. Parenchymal opacification was still present in the right cranial lung lobe (*) and occasionally in the right middle, caudal and accessory lung lobes (arrows). No lesion was appreciated in the left lung

Five months later the patient returned to the VHC for hematuria with normal respiratory character. She was receiving 0.84 mg/kg prednisolone q24h. Weight gain (0.73 kg) and mildly increased bronchovesicular sounds were appreciated. Urinalysis demonstrated hematuria, concentrated urine (specific gravity 1.071) and a quiet sediment. Anesthesia was performed with butorphanol (0.5 mg/kg IV) for premedication, followed by propofol (6 mg/kg IV) for induction. Anesthetic maintenance was performed using a propofol constant rate infusion (0.3 mg/kg/min). Thoracic CT documented near complete resolution of previously noted lung patterns compared to the prior CT (Fig. 3). A blind BAL showed 82% macrophages, 10% small lymphocytes, 3% mast cells, 2% non-degenerate neutrophils, 2% globule leukocytes and 1% eosinophils with a negative bacterial culture. Abnormal urinary signs were attributed to feline idiopathic cystitis. Amoxicillin/clavulanate was discontinued and the prednisolone dose was further reduced to 0.42 mg/kg PO q24h.

At a VHC recheck 2 months later, the cat lacked respiratory signs. Because selamectin (Revolution; Zoetis US), originally prescribed by the primary veterinarian, was inconsistently administered, feline heartworm antibody and heat-fixed occult heartworm ELISA were performed and were negative. Repeated echocardiography documented a functionally and structurally normal heart. Prednisolone was decreased to 0.23 mg/kg PO q24h and discontinued approximately 1 month later. The cat remained asymptomatic for respiratory signs.

## Discussion and conclusions

The current case report describes a cat with clinical and CT features of IPF that subsequently had complete resolution of CT lesions previously reported as compatible with fibrosis, and a clinical cure with empiric treatment and time. In humans, multidisciplinary collaboration between clinicians, radiologists and pathologists allowed development of high-resolution CT criteria as a surrogate for biopsy-confirmed IPF diagnosis [1]. These criteria focus on a CT UIP pattern including subpleural and basal predominance; reticular pattern; honeycombing with or without traction bronchiectasis; and absence of features inconsistent with UIP pattern (eg, predominant peribronchovascular lesions, ground glass abnormalities, micronodules or cysts, mosaic attenuation due to air trapping, and consolidation) [1]. In cats, thoracic CT features of fibrotic lung disease have been described infrequently, [4, 6, 7] are diverse, and likely represent different diseases culminating in fibrosis rather than a single disease analogous to human IPF. CT features of feline fibrotic lung disease confirmed by histologic examination include a reticular pattern [4, 7], subpleural interstitial thickening [4, 5, 7], architectural distortion [4], traction bronchiectasis [4, 7], honeycomb cysts [4, 7], ground glass opacities [4] and consolidation [5]. In dogs with IPF, CT scans show reticular abnormalities, traction bronchiectasis, honeycombing and ground-glass opacification [8–11]. A recent study suggests awake CT scans are adequate to diagnose IPF in West Highland White Terriers, a predisposed breed [12]. Worthy of note is that CT patterns of increased attenuation in both dogs and cats are inconsistent with a CT UIP diagnosis in humans, suggesting differences between species [1].

Other CT lesions may provide clues to the underlying disorder driving fibrotic change, for example, bronchiolar disease with bronchiolectasis and tree-in-bud patterns have been suggested to lead to airway-centered interstitial fibrosis [5]. Contrast-enhancing pulmonary masses have also been described [6]. Importantly, using CT criteria developed from a small number of cats with suspect IPF to diagnose this condition represents a dangerous precedence, especially given the aforementioned diversity in CT lung patterns. This is highly clinically relevant as feline IPF is considered end-stage with a grave prognosis and no viable treatments. Incorrect diagnosis using CT in lieu of lung biopsy could lead to a decision of euthanasia. Additional study of CT patterns of disease with confirmed diagnoses that mimic fibrotic lung disease is greatly needed.

Several aspects of this case, such as initial clinical signs, physical examination findings, lack of treatment response initiated by the primary care veterinarian, disease progression and initial CT interpretation fit with the diagnosis of IPF. However, in retrospect, the very young age of the cat was highly unusual. Although young cats can be affected with IPF, cases are nearly universally middle-aged or older cats [3, 6]. In particular, infectious diseases should be considered in young cats; thus, this cat was tested and treated empirically for helminths and Toxoplasmosis. Given the suspected poor prognosis, prednisolone was also administered in case of primary inflammatory disease. The majority of bronchiolar changes on CT [5] were not present in the original CT report but were identified on review by the coauthors (peribronchiolovascular thickening, bronchiolectasis, tree-in-bud and subpleural interstitial thickening). Historically, these patterns have been considered inconsistent with UIP/IPF in humans prompting consideration of another disorder [1]. Resolution of clinical signs and near complete resolution of thoracic CT lesions are also inconsistent with IPF.

**Fig. 4:**
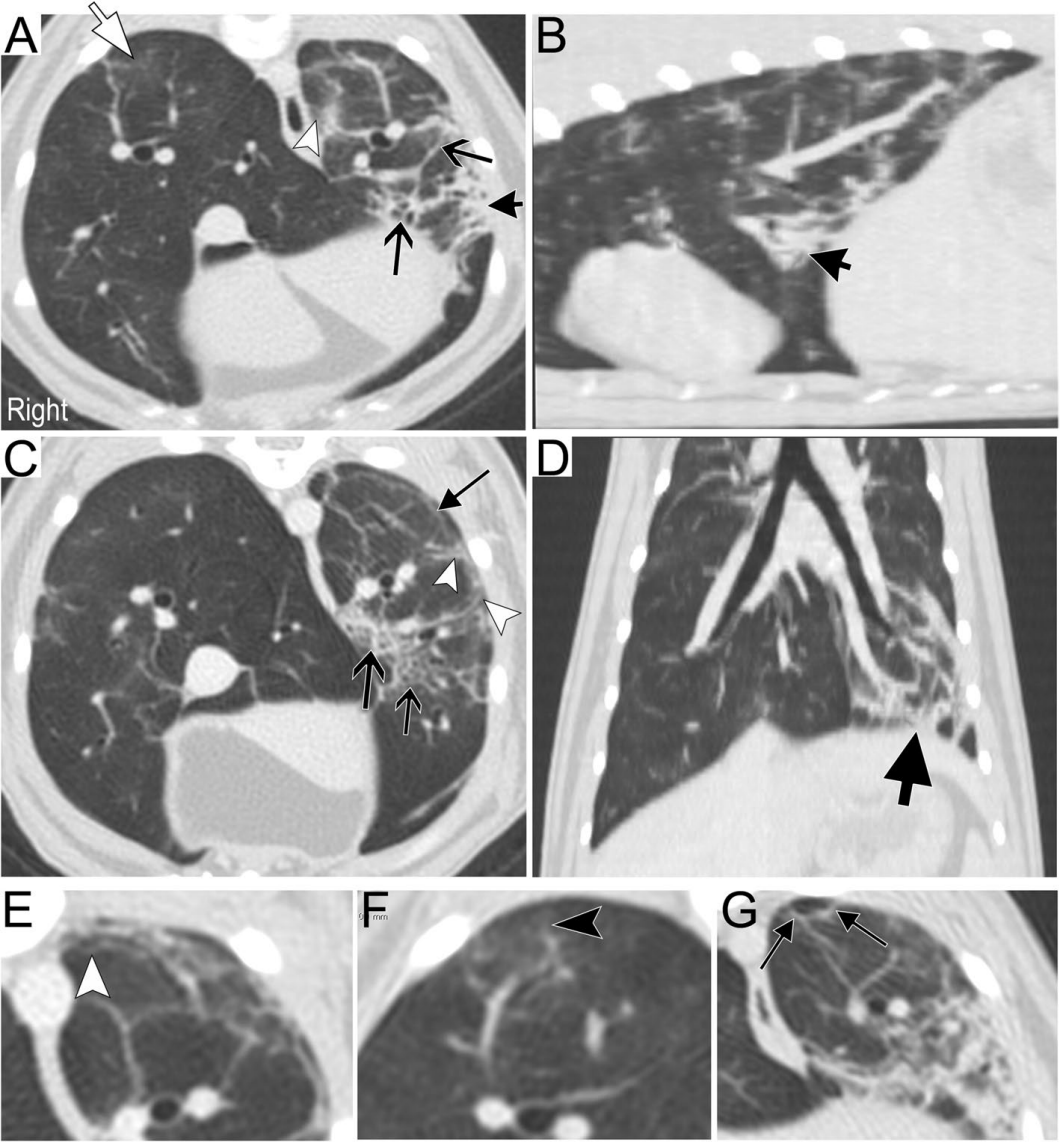
Computed tomographic (CT) images of two cats experimentally infected with *Toxocara cati*. Transverse (**A**, **F**, **G**) and multiplanar reconstructed sagittal (**B**) and dorsal (**D**) images are from cat 1 and transverse images (**C**, **E**) belong to cat 2. Both cats presented lesions categorized as parenchymal opacification (ground glass opacity (white arrow), consolidation (short arched black arrows)) and linear and reticular opacities (V-shaped arrows) including subpleural lines (small triangle-shaped black arrows), bronchiolectasis (white arrowheads) and tree-in-bud pattern (black arrowhead). The left lung was more severely affected than the right in both cats. DICOM studies courtesy of AR Dillon and TM Lee-Fowler

The young age at onset, initial CT lesions (Fig. 2) and mild airway eosinophilia, followed by resolution of clinical signs and CT lesions/airway eosinophilia (potentially in response to anthelmintic/anti-inflammatory therapy) of this cat fit with pulmonary migration of *Toxocara cati*, making it the authors’ top differential. The cat was acquired from a shelter with signs of respiratory disease. A high prevalence of *T. cati* in the feline population has been documented with identification of helminths in 53% of feral cat necropsies [13] and ova in 21 and 18% of feral and pet cats, respectively [14]. The parasite migrates through lungs and other tissues of infected cats before maturation within the small intestines, [15] making fecal examination imperfect for diagnosis. Clinical signs associated with parasitic migration through the lungs is not well described in cats, perhaps because of challenges with definitive diagnosis associated with the prepatent period. In one study, cats experimentally infected with *T. cati* lacked clinical signs despite airway eosinophilia; however, as it was an acute model, these authors caution that the long-term clinical effects could not be predicted [16]. Quantitative measures of lung density on CT images identified a significant increase in attenuation in infected versus uninfected cats [16]. However, there was no difference in CT scores between treated and untreated infected cats, and severe lung pathology was found even in the absence of adult parasites in the intestines [16]. Fig. 4 depicts CT images from two cats in that study showing similar/overlapping CT lesions to our cat especially linear opacities and bronchiolectasis. Peribronchovascular bundle thickening could reflect disease of small airways or vessels as these structures run in parallel to the lung periphery. Inflammation and medial hyperplasia of pulmonary arteries are seen in the lungs of cats infected with *T. cati* [17].

Other differential diagnoses for the clinical signs, thoracic imaging results and inflammatory BAL include Heartworm-Associated Respiratory Disease (HARD), *Toxoplasma gondii* or another type of parasitic infection. HARD refers to pathology of the airways, interstitium and pulmonary arteries induced by a host immune response to immature adult heartworms [18]. Importantly, cats with HARD frequently test antigen negative and antibody responses may be transient [18]. Thus, the negative antigen and antibody tests in the cat of our report would not definitively rule out HARD. Thoracic CT scans in cats with experimental HARD focused on bronchial and arterial measurements and ratios, [19] not on global changes including the interstitium which is the compartment most directly reflecting changes associated with IPF. In addition to HARD, toxoplasmosis was considered an unlikely but treatable differential in a young cat. There is a paucity of descriptions of thoracic radiographic changes in cats with pulmonary toxoplasmosis in the literature. Findings in 4 cats include nodular patterns, patchy mixed interstitial and alveolar patterns, diffuse bronchointerstitial patterns and pleural effusion [20–22]. A single case report of a thoracic CT in a cat with pulmonary toxoplasmosis showed a nodular pattern [23]. As these 5 cats are unlikely to represent the full spectrum of imaging lesions of feline pulmonary toxoplasmosis, the differential remained on our list. Negative toxoplasma-specific IgM titers made acute, active infection unlikely, but the single high IgG titer (1:2560) could have been consistent with chronic infection. Treating with clindamycin was opted in lieu of additional confirmatory testing. Finally, it is possible that another unidentified lungworm or migratory helminth that responded to fenbendazole may have been responsible for the clinical picture.

In conclusion, CT pulmonary patterns consistent with IPF in cats may not be adequate for diagnosis of this end-stage disease. This case illustrated reversibility of clinical signs and pathologic features which ruled out IPF and serves as a warning to prompt careful consideration of disease mimics and empirical treatment prior to recommendations of euthanasia. Additional multidisciplinary collaboration involving veterinary clinicians, radiologists and pathologists will be needed to establish CT-histologic correlates and determine under which conditions CT might serve as a useful surrogate for lung biopsy.

## Data Availability

Data sharing is not applicable to this article as no datasets were generated or analysed during the current study.

## Abbreviations

BAL: Bronchoalveolar lavage
CBC: Complete blood count
CT: Computed tomography
HARD: Heartworm associated respiratory disease
IPF: Idiopathic pulmonary fibrosis
IV: Intravenous
PO: Per os
SC: Subcutaneous
UIP: Usual interstitial pneumonia
VHC: Veterinary Health Center
